# Network analysis of maternal parenting practices and adolescent mental health problems: a longitudinal study

**DOI:** 10.1186/s13034-024-00728-w

**Published:** 2024-03-19

**Authors:** Xinlu Sun, Ting Yuan, Feifei Chen, Yan Li, Nengzhi Jiang

**Affiliations:** 1 School of Psychology, Shandong Second Medical University, 7166 Baotong West Street, Weifang, Shandong 261053 China; 2 Department of Psychology, DePaul University, 2219N Kenmore Ave, Chicago, IL 60614 USA

**Keywords:** Maternal parenting practices, Adolescent mental health, Contemporaneous network, Temporal network

## Abstract

**Background:**

An extensive literature has shown a strong connection between maternal parenting practices and adolescent mental health problems. However, it has been difficult for previous research to map a dynamic concurrent and prospective relationships within and between types of parenting practices and adolescent mental health problems. The present study addressed these issues using a network analysis approach and a longitudinal design.

**Methods:**

This study involved 591 Chinese adolescents (249 males; mean age at T1 = 13.53) and their mothers (mean age at T1 = 39.71) at two time points (T1 and T2) with eighteen months apart. Mothers reported their parenting practices including warmth, monitoring, inductive reasoning, hostility, and harshness, while adolescents reported their mental health problems including anxiety, depression, aggression, and conduct problems. Network analysis was conducted for contemporaneous networks at T1 and T2 and temporal networks from T1 to T2.

**Results:**

The contemporaneous networks revealed the negative association between monitoring and conduct problems served as the main pathway through which parenting practices and adolescent mental health mutually influenced each other, and further, warmth was the most influential parenting practice on adolescent mental health. The temporal network revealed that maternal hostility exerted the most influence on adolescent mental health problems, whereas adolescents’ depression was most influenced by maternal parenting practices. Moreover, maternal hostility was most predicted by maternal harshness.

**Conclusions:**

This study presents a novel perspective to gain a better understanding of the dynamics between and within maternal parenting practices and adolescent mental health problems. Findings highlight maternal harshness and warmth as potential prevention and intervention targets for adolescent mental health problems.

**Supplementary Information:**

The online version contains supplementary material available at 10.1186/s13034-024-00728-w.

## Introduction

Extensive research has examined various influences on adolescents’ mental health problems [1, 2]. According to the Ecological Systems Theory, the developing children’s interactions with parents, as the one of the core aspects of the microsystem, impact every aspect of their development [3]. Supporting this theory, parenting practices have been shown to have an immediate effect on adolescents’ mental health problems [4–6]. Despite these research advances, a thorough comprehension of the intricate interplay between distinct parenting practices and adolescent mental health problems awaits to be further explored, which may be useful in future potential prevention and intervention efforts to promote adolescent mental health.

Considerable evidence has accumulated to suggest distinct parenting practices associated with different adolescent mental health problems. With respect to externalizing problems, empirical evidence indicates that negative parenting behaviors (e.g., harshness, hostility) are positively associated with more adolescent aggression and conduct problems [7–9]. In contrast, positive parenting behaviors (e.g., warmth, inductive reasoning, monitoring) show a negative association with these issues [10]. With regard to internalizing problems, such as anxiety and depression, research has shown that negative parenting behaviors (e.g., harshness, psychological control, authoritarian) are associated with more internalizing problems, while positive parenting behaviors (e.g., warmth, monitoring, autonomy granting, and authoritative parenting) relate to fewer internalizing problems [6, 11, 12]. In addition to these relationships, past research has also shown that distinct parenting practices influence each other [13–15], and the same is true for the different adolescent mental health problems [16, 17].

Although there is extensive evidence addressing the relationships between parenting practices and adolescent mental health problems, a simultaneous presentation of the link within the domain of either parenting practices or the adolescent mental health problems and of the prominent relationships between specific parenting practices and specific adolescent mental health problems has been a challenging task. The distinct parenting practices and adolescent mental health problems may possess an interconnectedness quality. Therefore, an analytic approach that simultaneously accounts for these complex interconnections among these two sets of constructs is greatly needed.

In comparison to the latent variable framework, the network approach to psychopathology posits that alterations in one symptom are hypothesized to propagate within the system, resulting in consequential modifications in other symptoms [18, 19]. The network analysis, a method developed from Borsboom’s network theory of psychopathology [20], allows for the inclusion of all variables at the same time for analysis and visualization of the connections of them as a network [18]. The nodes in this network represent the components of research variables in the model, and the edges represent the dynamic relationships between the components of research variables, thus allowing us to visualize more intuitively how distinct parenting practices and adolescent mental health problems interact with each other [21]. Furthermore, the importance of a node in the network can be estimated by the centrality indices [22]. A node with high centrality indicates that it is more connected to other nodes or research variables and therefore, it can be considered a central variable in the network.

The network analysis has only recently received attention in parenting research. So far, there are only two studies investigated the network of parenting practices and adolescent mental health problems. In one of the studies, monitoring behavior was the most influential node with high levels of centrality in the parenting network [23]. This study also showed the association of poor monitoring with conduct problems served as the primary pathway through which parenting behavior and adolescent mental health mutually influenced. The other study found that harshness was directly related to higher levels of conduct problems [24]. However, these two studies estimated contemporaneous networks using cross-sectional data, which can only provide limited information about the temporal and causal relationships between distinct parenting practices and adolescent mental health problems.

The cross-lagged panel network (CLPN) model was developed to estimate the temporal effects between individual components of a construct in panel data [25]. The advantage of CLPN is that the role of each component can be systematically analyzed, and the longitudinal predictive pathway of each component can be estimated, thereby deepening our understanding of the connections between parenting practices and adolescent mental health problems [26]. In addition, CLPN divides node centrality into out-prediction (affecting other nodes) and in-prediction (being affected by other nodes), with nodes with high out-prediction centrality being more likely to influence the entire network and serving as potential intervention targets [27]. Given that parent practices and adolescent mental health problems are not just concurrently associated [23, 24], but also longitudinally influenced each other [7, 12]. Therefore, a longitudinal design using the CLPN is greatly needed to gain a better comprehension of the temporal relationships between distinct parenting practices and adolescent mental health problems.

Due to the fact that mothers play a more important role than fathers in raising their children in Chinese families [28, 29], the present study examined the contemporaneous relations of five distinct maternal parenting practices (harshness, hostility, warmth, inductive reasoning, and monitoring) and four types of adolescent mental health problems (anxiety, depression, aggression, and conduct problems) and temporal relations between these two sets of constructs at two time points. Specifically, we first constructed two contemporaneous networks at T1 and T2 to reveal the most influential components (the central nodes) in the network and the primary pathway through which the two sets of constructs influenced each other. Next, we constructed a temporal network to investigate the direction and weights of influence between and within the two constructs and to identify which components exerted higher influence on other components (high out-prediction) and were most influenced (in-prediction). Recent studies among Chinese adolescents have revealed that parental warmth and harshness exert predictive influence on a wide range of mental health problems, particularly on depressive symptoms [30–33]. Specifically, parental warmth has been found to be a vital protective factor, whereas harshness serves as a significant risk factor. Based on these findings, we hypothesized that maternal warmth and harshness would be the most influential parenting practices, whereas adolescent depression would be the most influenced mental health problems.

## Method

### Participant and procedure

We used a convenience cluster sampling method to recruit 642 Chinese adolescents and their mothers in Beijing, including 196 primary school students (Mean age = 11.01 years, SD = 0.96, 110 females), 294 junior high school students (Mean age = 14.00 years, SD = 1.03, 164 females), and 104 senior high school students (Mean age = 16.73 years, SD = 0.73, 63 females). These adolescents were instructed by trained research assistants to complete the relevant checklist about their mental health problems in classroom during school hours. Mothers’ questionnaires were taken home by the adolescents to complete and sent back to research assistants.

At Time 1, adolescents were on average 13.62 years old (SD = 2.25, ages ranged 8–17 years, 362 females) and mothers were on average 40.04 years old (SD = 4.25, ages ranged 29–56 years). The minority of mothers (4.6%) had a primary school education or less, the majority of the mothers (59.2%) had a middle or high school degree, about one third of them (29.9%) had a university or junior college degree, and the remaining (6.3%) were missing. In relation to monthly family income, about one-fifth (21.8%) reported less than ¥3000, half (49.2%) between ¥2000 and ¥6000, a minority (14.6%) between ¥6000 and ¥10,000, a tiny minority (11.0%) more than ¥10,000, and the remaining (3.4%) were missing. Furthermore, the majority of adolescents (89.4%) were in two-parent families, the minority (7.1%) were others, and the remaining (3.5%) were missing. After 18 months (T2), we collected data from 591 adolescents (249 males and 342 females, attrition rate = 7.9%) who participated in T1 data collection and their mothers using the same procedure. Attrition occurred on the adolescents who did not come to school on the day of data collection due to leave of absence, change of school, and other reasons.

This study was approved by the corresponding author’s Institutional Review Board (Protocol Number: 2021YX027). Prior to the survey, we provided school administrators, students, and their mothers with a written informed consent that outlined the objectives of this study, the procedures for ensuring data security, the voluntary nature of participation, and their right to withdraw their involvement at any point. Written consent was obtained from participants and school administrators. Participants received feedback on maternal parenting practices and adolescent mental health problems based on their responses to questionnaires.

### Measurements

#### Maternal parenting practices

Mothers completed a 32-item measure regarding their parenting practices during the past 12 months with a 5-point scale ranging from 1 (never) to 5 (always). This measure was originally developed for the Iowa Youth and Families Project [34–36] that has been shown to capture important parenting dimensions in Western countries [37, 38]. A Chinese version of four subscales (inductive reasoning, warmth, harshness, hostility) has been validated in several studies among Chinese populations and established good reliabilities, which were used directly in this study [39, 40]. Two additional subscales (monitoring and consistent discipline) used in previous studies were translated into Chinese through the translation and back-translation process [37, 38]. Among these items, six parenting practices were assessed using these measures, including warmth (e.g., express warmth and support to children, 8 items), hostility (e.g., yell, insult or be angry to children, 6 items), monitoring (e.g., know where children are, 6 items), consistent discipline (e.g., discipline children according to mood, 4 items), harshness (e.g., hit or spank children, 3 items), and inductive reasoning (e.g., discipline children with reasoning, explaining and talking, 5 items). Consistent discipline was not included in the analysis because the Cronbach’s alpha was less than 0.60 at two time points. For the other five parenting practices in this study’s sample, Cronbach’s alphas range from 0.59 to 0.87 at T1 and 0.67 to 0.88 at T2 (Table 1).

**Table 1:** Skewness, kurtosis and Cronbach’s alpha of study variables

Node	T1			T2		
	Skewness	Kurtosis	Cronbach’s alpha	Skewness	Kurtosis	Cronbach’s alpha
Anxiety	0.42	-0.08	0.86	0.34	0.04	0.87
Depression	0.14	-0.59	0.77	0.94	0.54	0.85
Aggression	0.97	0.69	0.82	1.16	1.46	0.84
Conduct problem	1.82	6.19	0.65	2.31	9.62	0.74
Warmth	-0.65	0.30	0.87	-0.44	-0.18	0.87
Monitoring	-1.49	2.90	0.87	-1.38	2.33	0.88
Hostility	1.15	2.16	0.83	1.10	2.44	0.84
Inductive reasoning	-0.79	0.53	0.82	-0.60	0.06	0.85
Harshness	1.80	6.82	0.59	1.91	6.59	0.67
Consistent discipline	0.63	0.29	0.50	0.59	0.09	0.54

#### Depression

Adolescents’ depression was measured by the Chinese version of the Children’s Depression Inventory (CDI) [41, 42], a 27-item measure with a 3-point Likert scale (e.g., 0 = I hate myself, 2 = I don’t like myself, 3 = I like myself). Adolescents reported their depressive mood and behaviors in the past two weeks. The Chinese version of the CDI had been established good reliability and validity in previous studies [43, 44]. The Cronbach’s alphas of the CDI at two time points in this study were 0.77 and 0.85, respectively.

#### Anxiety

The trait subscale of the State-Trait Anxiety Inventory (STAI-Y) was used to measure adolescents’ anxiety in the past two weeks [45]. This measure included 20 items with a 4-point Likert scale (from 1 = never to 4 = almost always). The Chinese version of the STAI had demonstrated good reliability and validity [46]. The Cronbach’s alphas for anxiety at two time points in this study were 0.86 and 0.87, respectively.

#### Externalizing problems

The Youth Self-Report Scale (YSR) was used to assess two externalizing behaviors, aggression and conduct problems, in adolescents in the past 6 months [47]. The Chinese version of this scale had been widely used in previous studies [48], and Cronbach’s alphas ranged from 0.65 to 0.82 at T1 and from 0.74 to 0.84 at T2 (Table 1).

### Analytic approach

All data screening and descriptive statistics were performed in SPSS 26.0. Table 1 presents the skewness, kurtosis and Cronbach’s alphas of study variables at T1 and T2. Furthermore, we plotted the correlation matrix between the variables using the R package ‘corrplot’ in R Version 4.1.3 [49]. We calculated the residuals of the variables after controlling for the covariates (adolescents’ gender, age, mother’s education status, and family monthly income), and then used the residuals to run the network analyses.

All network analyses were conducted in R. We first estimated the cross-sectional network of the Gaussian graphical model (GGM) [50]. Specifically, we used the ‘estimateNetwork’ function in R package ‘bootnet’ [22] to estimate the network and then the ‘averageLayout’ function in the R package ‘qgraph’ [51] to lay out the network, which fixed the same nodes in both networks at the same location for visual comparison. The temporal network was estimated using CLPN analysis [52]. We computed the unstandardized and regularized autoregressive coefficients within (undirected) and between time points using the R package ‘glmnet’ [53] and visualized them using the R package ‘qgraph’. To further estimate sparser and interpretable networks, we used the least absolute shrinkage and selection operator (LASSO), which can accurately shrink small /negligible edge coefficients to zero, thus reducing the possibility of edge false positives [22, 54].

Next, we used R package ‘bootnet’ to compute centrality indices to determine the importance of each node. For contemporaneous networks, we calculated expected impact (EI; sum of edge weights from this node to all other nodes) [55]. EI is more suitable than Strength (sum of the absolute values of edge weights from this node to all other nodes) for networks in this study since both the edge weights and the positive and negative directions of the edges are considered [56]. The higher the values of EI, the stronger the impact of this node in the network. For temporal network, we calculated the in-prediction and out-prediction for cross-lagged (excluding autoregressive path of the node of interest) and cross-construct (excluding autoregressive path and paths within the same construct) [57]. The in-prediction refers to the proportion of variance at a node of T2, which is accounted for by the nodes of T1. The higher the in-prediction, the more it is influenced by other nodes. The out-prediction refers to the effect of a T1 node on the T2 nodes [25], and the higher it is, the more it influences the other nodes.

Finally, we estimated the edge weight accuracy by calculating the 95% confidence intervals (CIs) around each edge weight value using 1000 iterations with nonparametric bootstrapping [22]. Narrow 95% CIs suggest that the edge weights have good accuracy. Besides, we estimated the correlation stability (CS) coefficient using case-drop bootstrapping to determine the stability of the centrality indices [22]. The CS coefficient should not fall below 0.25 and, ideally, should exceed 0.50. We next conducted a bootstrap difference test on the edge weights and centrality indices to examine whether there was a significant difference between them [22].

## Results

### Descriptive statistics and correlation analysis

**Fig. 1:**
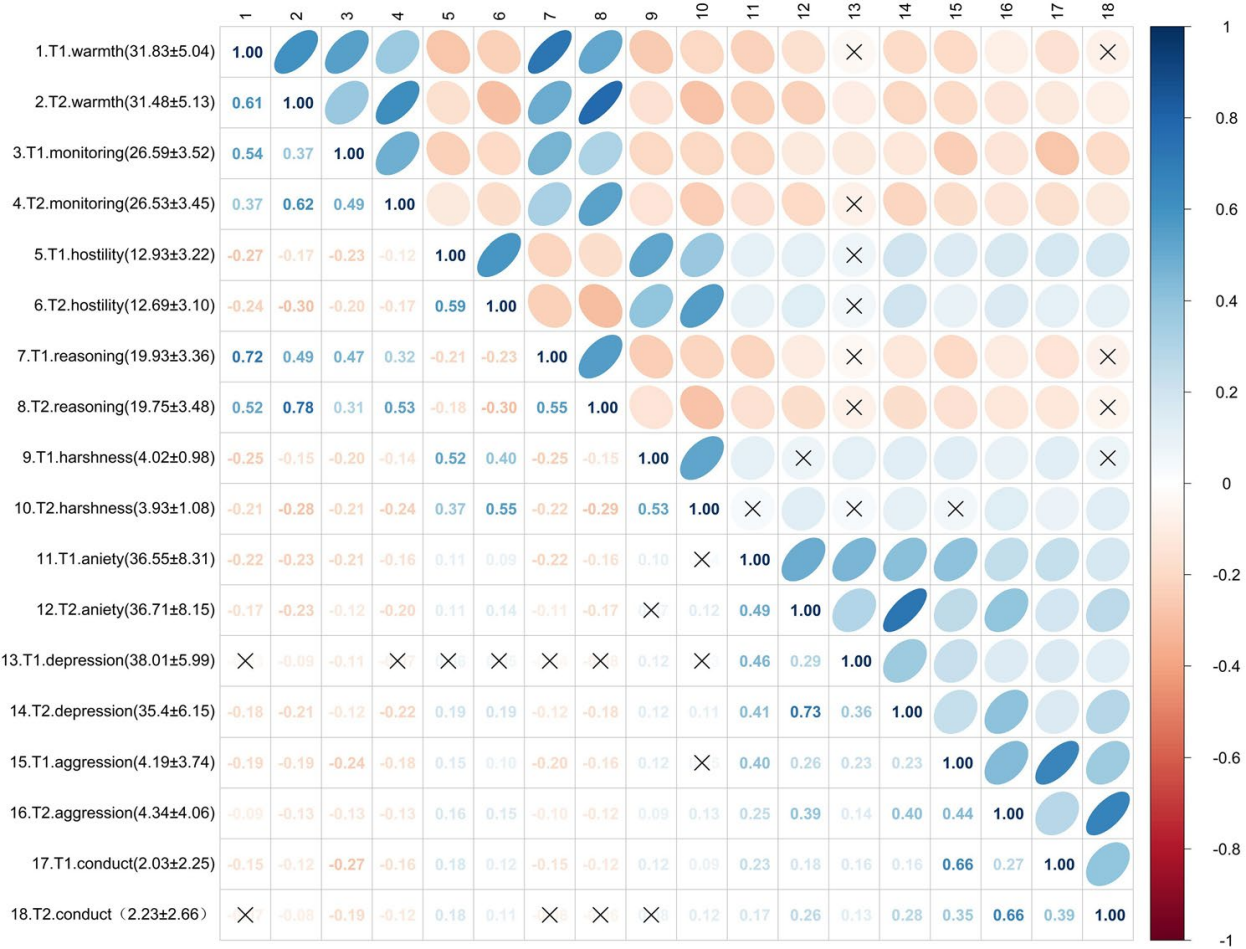
Descriptive statistical results and correlation matrix for maternal parenting practices and adolescent mental health problems. The color and direction of the ellipse represents the direction of the correlation, where blue, top-right to bottom-left direction corresponds to a positive correlation and red, top-left to bottom-right direction corresponds to a negative correlation. The color shade and shape of the ellipse represent the strength of the correlation, where darker colors and flatter ellipses correspond to stronger correlations. “×” represents the correlation coefficient corresponding to *p* > 0.05. Numbers in the parentheses next to the variable names represent the means and standard deviations for the corresponding variables

The variables in this study generally conformed to the assumption of normality (see Table 1). Figure 1 presents the descriptive statistics (mean and standard deviation) and the correlation matrix for the five dimensions of maternal parenting practices and the four types of adolescent mental health problems. The results showed that there were significant correlations among most of the variables. Among them, the degree of positive correlation ranges from 0.11 to 0.78, and the degree of negative correlation ranges from − 0.30 to -0.08.

### Contemporaneous networks

**Fig. 2:**
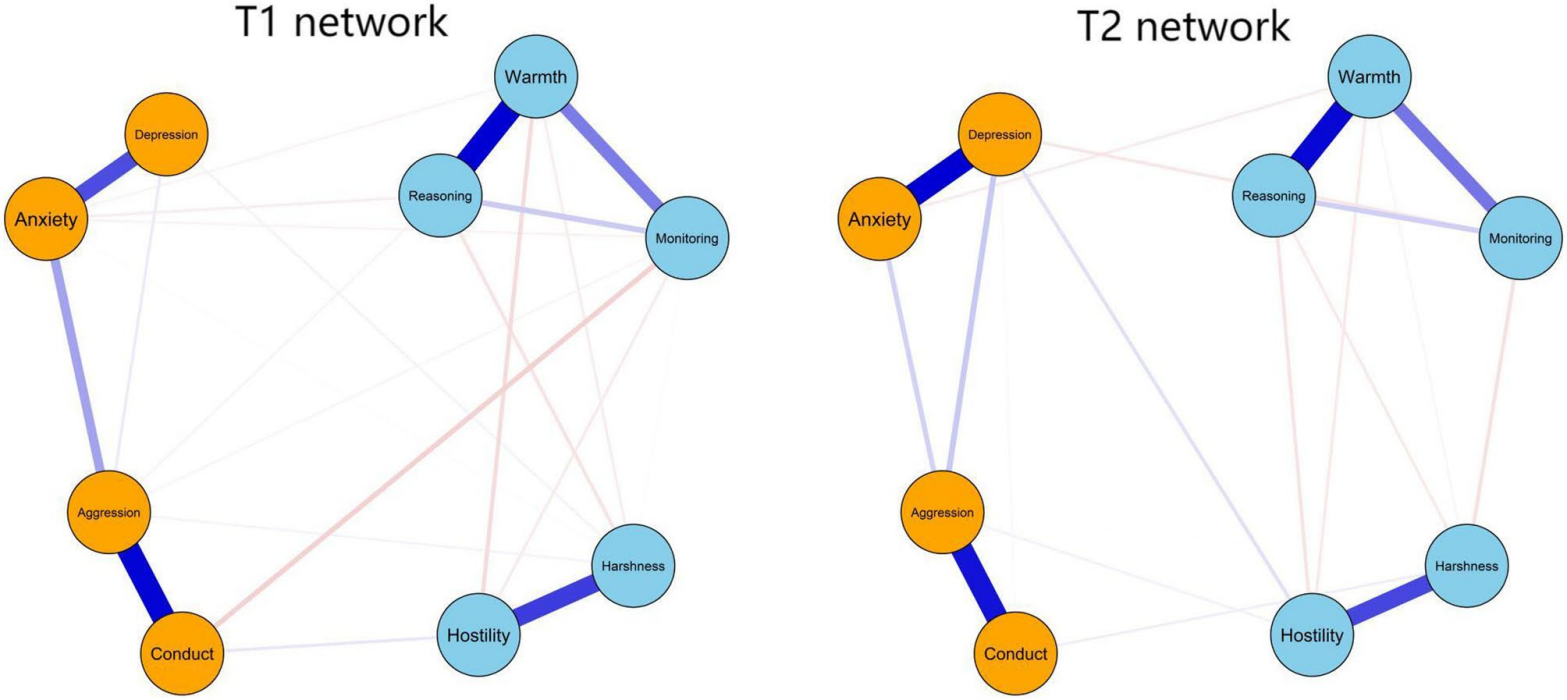
Contemporaneous networks of maternal parenting practices and adolescent mental health problems. Blue nodes represent maternal parenting practices, orange nodes represent adolescents’ mental health problems. Blue lines indicate positive relations, whereas red lines indicate negative relations. The thicker the lines are, the stronger the association

**Fig. 3:**
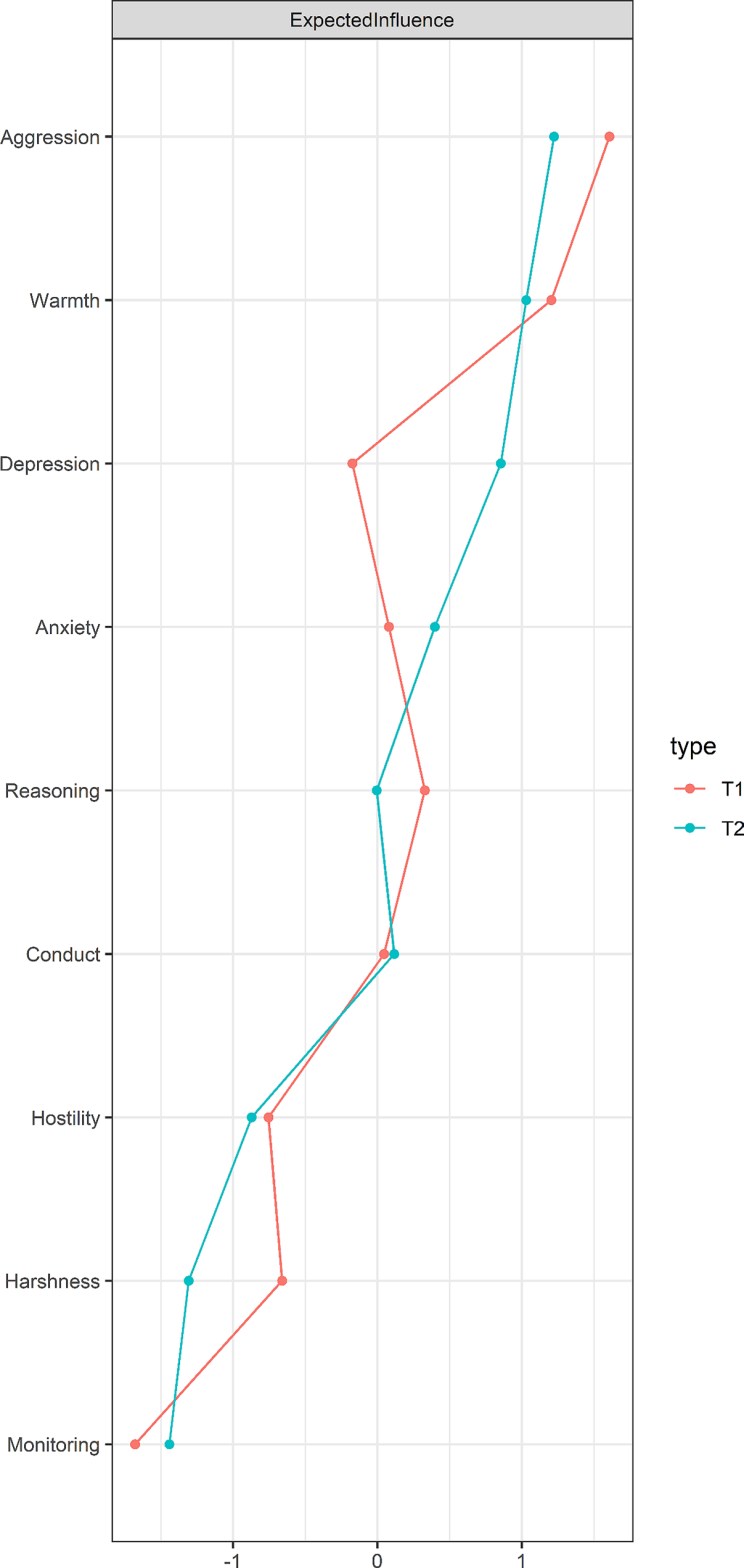
Centrality indices of maternal parenting practices and adolescent mental health problems. Red and blue lines are for T1 and T2 variables, respectively

The contemporaneous network analysis results at both time points revealed positive associations within maternal positive (warmth, inductive reasoning, and monitoring) and within negative (harshness, hostility) parenting practices, as well as within adolescent mental health problems (anxiety, depression, aggression, and conduct problems) (see Fig. 2; Table 2). Within the two constructs, the strongest edges were warmth-inductive reasoning (average *r* = 0.59), aggression-conduct problem (average *r* = 0.57), anxiety-depression (average *r* = 0.51), and hostility-harshness (average *r* = 0.45). Across the two sets of constructs, the strongest edge was between maternal monitoring and adolescent conduct problems (average *r* = -0.05). In addition, maternal parenting practices with the highest centrality was warmth (average EI = 1.12; see Table S1), while adolescent mental health problems with the highest centrality was aggression (average EI = 1.42), as shown in the Fig. 3. These findings indicate the strong impact of maternal warmth and aggression in the network.

**Table 2:** Strongest undirected edges of contemporaneous networks

Edge	Undirected edge weight r		
	T1	T2	Average
Warmth-Inductive reasoning	0.57	0.62	0.59
Aggression-Conduct problem	0.56	0.59	0.57
Anxiety-Depression	0.39	0.63	0.51
Hostility-Harshness	0.43	0.46	0.45
Warmth-Monitoring	0.29	0.34	0.32
Anxiety-Aggression	0.20	0.11	0.16
Monitoring-Inductive reasoning	0.12	0.12	0.12
Depression-Aggression	0.04	0.13	0.09
Hostility-Depression	0.00	0.08	0.04
Hostility-Conduct problem	0.05	0.00	0.02
Harshness-Conduct problem	0.00	0.04	0.02
Hostility-Aggression	0.00	0.03	0.01
Harshness-Aggression	0.02	0.00	0.01
Harshness-Depression	0.03	0.00	0.01
Depression-Conduct problem	0.00	0.02	0.01
Monitoring-Aggression	-0.02	0.00	-0.01
Inductive reasoning-Aggression	-0.02	0.00	-0.01
Monitoring-Anxiety	-0.03	0.00	-0.01
Inductive reasoning-Anxiety	-0.04	0.00	-0.02
Monitoring-Hostility	-0.04	0.00	-0.02
Monitoring-Depression	0.00	-0.06	-0.03
Warmth-Harshness	-0.04	-0.02	-0.03
Hostility-Inductive reasoning	0.00	-0.07	-0.03
Warmth-Anxiety	-0.03	-0.05	-0.04
Monitoring-Harshness	-0.01	-0.07	-0.04
Monitoring-Conduct problem	-0.10	0.00	-0.05
Inductive reasoning-Harshness	-0.06	-0.05	-0.05
Warmth-Hostility	-0.08	-0.05	-0.07

The table only shows the average of the edge weights with absolute values stronger than 0.01 at either T1 or T2 or both

Furthermore, the small to moderate CIs around edge weights suggest the contemporaneous networks had moderate to strong edge weight accuracy (see Fig. S1), and high CS coefficient for EI (CS at T1 = 0.67, CS at T2 = 0.75) indicated strong stability for centrality indices. Edge weight difference tests and centrality difference tests are shown in Figs. S2 and S3.

### Temporal network

**Fig. 4:**
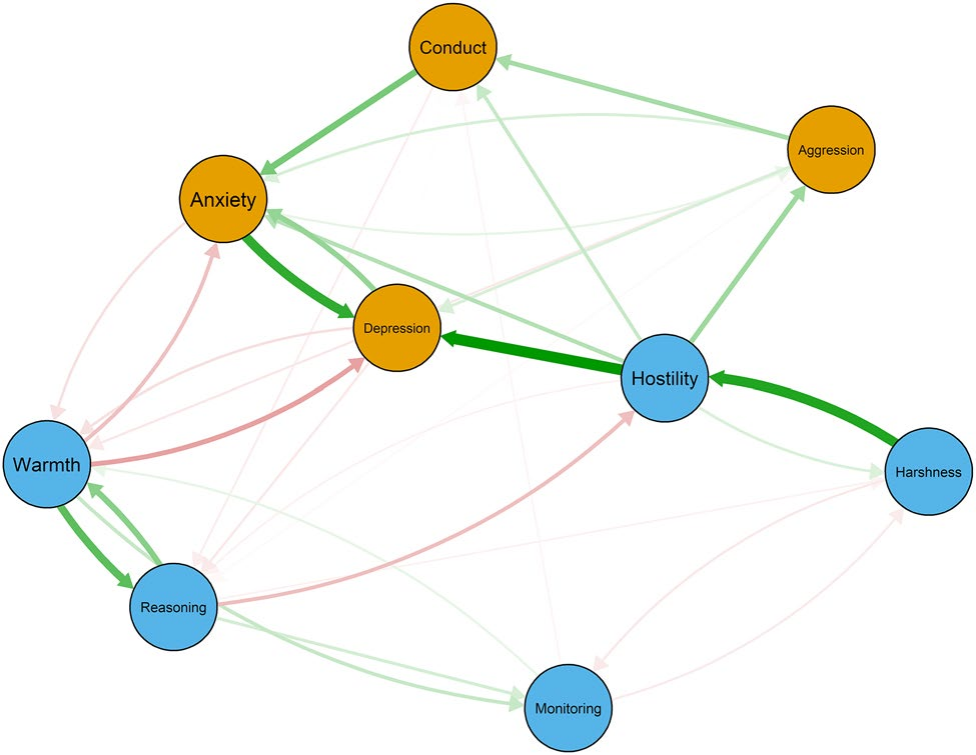
Temporal network of maternal parenting practices and adolescent mental health problems. Blue nodes represent maternal parenting practices, orange nodes represent adolescents’ mental health problems. Green lines indicate positive relations, whereas red lines signal negative relations, and line thickness reflects strength of associations

Figure 4 illustrates the temporal network without autoregressive paths, as autoregressive paths are too strong to visualize the cross-lagged paths well (see Fig. S4 for the temporal network containing autoregressive paths). The temporal network analysis results show that the strongest edge within maternal parenting practices was that maternal harshness at T1 positively predicted hostility at T2 (d = 0.20; see Table 3). Furthermore, maternal warmth and inductive reasoning also mutually predicted each other (Warmth→Inductive reasoning: d = 0.15; Inductive reasoning→Warmth: d = 0.11). Within adolescent mental health problems, the strongest edge was that adolescent anxiety at T1 positively predicted depression at T2 (d = 0.19; see Table 3). Notably, adolescent depression at T1 also positively predicted anxiety at T2 (d = 0.10; see Table 3). The strongest edges across the two sets of constructs were that maternal hostility at T1 positively predicted adolescent depression (d = 0.23) and aggression (d = 0.09) at T2. Additionally, maternal warmth at T1 negatively predicted adolescent depression (d = -0.08) and anxiety (d = -0.06) at T2.

**Table 3:** Strongest directed edges of temporal network from T1 to T2

T1 to T2 edge	Directed edge weight d
Hostility→Depression	0.23
Harshness→Hostility	0.20
Anxiety→Depression	0.19
Warmth→Inductive reasoning	0.15
Conduct problem→Anxiety	0.12
Inductive reasoning→Warmth	0.11
Depression→Anxiety	0.10
Hostility→Aggression	0.09
Aggression→Conduct problem	0.08
Hostility→Anxiety	0.07
Warmth→Monitoring	0.05
Hostility→Conduct problem	0.05
Inductive reasoning→Monitoring	0.04
Aggression→Depression	0.03
Aggression→Anxiety	0.03
Hostility→Harshness	0.03
Anxiety→Aggression	0.02
Monitoring→Warmth	0.02
Aggression→Inductive reasoning	-0.01
Monitoring→Conduct problem	-0.01
Hostility→Inductive reasoning	-0.01
Inductive reasoning→Harshness	-0.01
Conduct problem→Inductive reasoning	-0.02
Monitoring→Harshness	-0.02
Depression→Inductive reasoning	-0.02
Harshness→Monitoring	-0.02
Aggression→Warmth	-0.02
Anxiety→Warmth	-0.03
Depression→Warmth	-0.03
Warmth→Anxiety	-0.06
Inductive reasoning→Hostility	-0.06
Warmth→Depression	-0.08

The table only shows edges with absolute values of edge weights stronger than 0.01

**Fig. 5:**
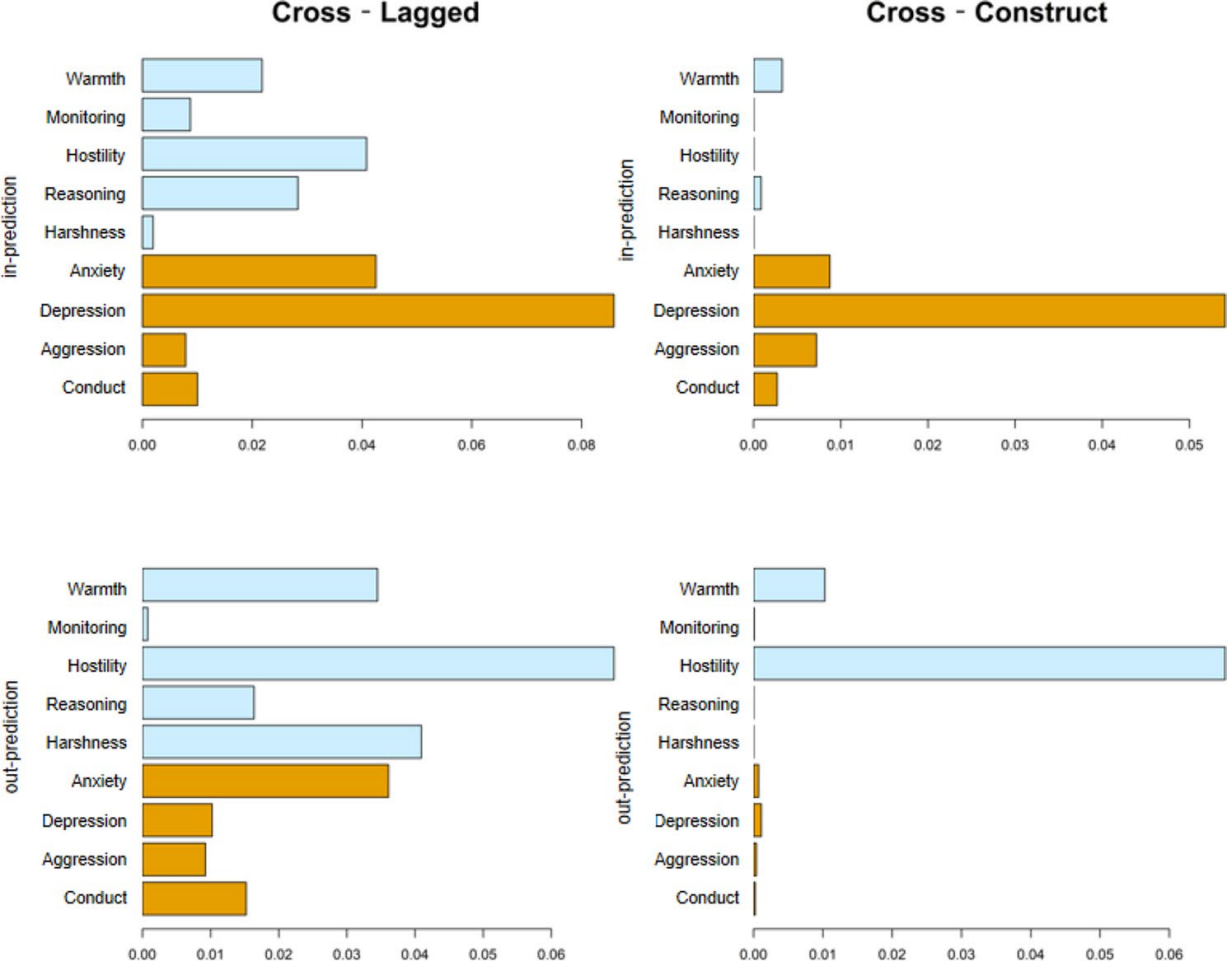
In-prediction and out-prediction for the cross-lagged (left) and cross-construct (right) of temporal network. Blue bars represent maternal parenting practices, orange bars represent adolescents’ mental health problem

Figure 5 shows the in-prediction and out-prediction estimates of cross-lagged (on the left) and cross-construct (on the right) analysis. The cross-lagged results showed that maternal harshness appeared to exert the most influence on other variables in the network due to the higher out-prediction and the lowest in-prediction estimates. With regard to adolescent mental health problems, depression appeared to be the most influenced component due to the highest in-prediction and low out-prediction estimates. However, because of the strong links within maternal parenting practices as well as within adolescent mental health problems, it was difficult to delineate the mutual influence between the two sets of constructs. Therefore, cross-construct analysis became especially informative. The cross-construct results indicated that hostility was the maternal parenting practice influenced adolescent mental health problems the most due to the highest out-prediction estimates, whereas depression was the adolescent mental health problem most influenced by maternal parenting practices due to the highest in-prediction estimates. Furthermore, both the in-prediction and out-prediction of the temporal network are highly stable (in-prediction CS = 0.52, out-prediction CS = 0.52).The results of bootstrap confidence intervals around edge weight and bootstrap difference tests are shown in Fig.S5-Fig.S7. Because some of the bootstrap difference tests were not significant, the results should be interpreted with caution.

## Discussion

The present study offered a novel perspective to the concurrent and prospective associations between five distinct maternal parenting practices and four types of adolescent mental health problems. Our study reveals that the negative correlation between maternal monitoring and conduct problems is the main pathway linking the two constructs, while hostility and warmth have the strongest impact on adolescent mental health problems. Notably, maternal hostility was most predicted by harshness. These findings provide valuable insights into the dynamic nature within maternal parenting practices and adolescent mental health problems as well as mapping the complex connections between the two sets of constructs, which may be useful in future potential prevention and intervention efforts to promote adolescent mental health.

Contemporaneous networks in our study revealed how distinct maternal parenting practices were uniquely associated with adolescent mental health problems. For instance, the link between maternal monitoring and adolescent conduct problems served as the main pathways through which the two sets of constructs mutually influenced, which was consistent with the cross-sectional network result conducted in a different cultural context [23]. A previous structural equation model research also suggested that parental monitoring can have an impact on delinquent behavior both directly and indirectly through contacts with delinquent peers [58]. It makes sense that adolescents who are not adequately monitored by their parents have more opportunities to socialize with problematic peers, who will likely encourage them to participate in conduct problems. According to delinquency theory [59], parental behavioral control, such as rule-setting, monitoring, and effective punishment, encouraged a child’s growth in self-control, which in turn prevented externalizing problems. However, we should be cautious interpreting these results as the contemporaneous networks preclude information about the direction of relationships between the components.

The temporal network provides insights into how maternal parenting practices and adolescent mental health problems mutually influenced each other over time. For example, harshness strongly impacted other maternal parenting practices, but did not directly impact adolescent mental health problems. Notably, the strongest edge in the maternal parenting practices was that harshness positively predicted hostility. While previous studies had found a link between parental harshness and adolescent mental health problems [7, 12, 24], our study extended this finding by demonstrating that harshness affects adolescent mental health problems by influencing other parenting practices, particularly hostility. On the contrary, hostility showed little impact on other maternal parenting practices, but a strong direct impact on adolescent mental health problems. We discovered that hostility positively predicted future adolescent depression and aggression. This is in accordance with Bandura’s social learning theory [60], parental hostility may be imitated by the child, which may consequently result in more externalizing problems. In addition, several studies have found that negative parenting practices (e.g., hostility, psychological control, and negative control) are negatively associated with emotion regulation, suggesting that hostile parenting may contribute to internalizing problems by affecting adolescents’ emotional regulation ability [28, 61, 62]. From an intervention perspective, our results indicated that harshness should be seen as a potential target to curtail hostility which predicted adolescent mental problems more directly.

Relatedly, maternal warmth played an important role in both contemporaneous and temporal networks. In particular, warmth had bidirectional relationships with inductive reasoning, indicating that these practices mutually influenced each other over time such that warmth predicted more inductive reasoning, which in turn predicted more warmth. Furthermore, warmth negatively predicted adolescent anxiety and depression. This may be due to the fact that mothers with warmth show their children more love and admiration, which can encourage positive feelings in children [63]. Additionally, parental support might reduce negative feelings in adolescents and lessen their chance of developing depression [64]. These results highlight the beneficial role of maternal warmth for parenting practices and adolescent mental health problems.

In addition, adolescent depression was influenced not only by maternal parenting practices but also by other mental health problems. For example, anxiety was identified as the most significant mental health factor influencing depression in the present study. This finding aligns with existing literature indicating that anxiety often precedes depression [65] and that these two conditions commonly co-occur in adolescents [66]. Moreover, aggression was also a strong predictor of depression. According to the developmental cascade model [67], externalizing behaviors may indirectly lead to internalizing problems via mediating variables. Adolescent aggressive behaviors, for instance, may trigger negative responses (such as punishment or rejection) from parents, teachers and classmates, which may then exacerbate adolescent’s other mental health problems. Notably, aggression also had a high centrality in the contemporaneous networks, which reminded us to give it more attention as well.

Our findings should be interpreted in light of several limitations. First, maternal parenting practices were reported only by mothers in our study. A previous meta-analysis has demonstrated associations between parenting and adolescent internalizing problems that were moderated by different informants [12]. Second, it has been argued that paternal coparenting plays a vital role in adolescent’s development [68]. Therefore, future research should integrate paternal parenting practices in the network analysis.

## Conclusions

To our knowledge, this is the first study used both contemporaneous and temporal network analysis to explore the relationship between maternal parenting practices and adolescent mental health problems. Results demonstrated the complex relationships between and within the two sets of constructs and revealed the most influential maternal parenting practices. Specifically, the negative association between monitoring and conduct problems was the main pathway in which parenting practices concurrently connected with adolescent mental health problems. Moreover, maternal warmth was the most protective factor for adolescent mental health problems. Conversely, maternal hostility, which was most predicted by maternal harshness, was the strongest risk factor for adolescent mental health problems. In addition, adolescents’ depression was most influenced among adolescent mental health problems predicted by maternal parenting practice. In summary, this study’s application of network modeling extends previous research by simultaneously considering various parenting practices and adolescent mental health problems in a dynamic interconnected system and further our results highlight the important roles of parent harshness, warmth, and adolescent depression in adolescents’ mental health from a novel network perspective. These findings may be of particular importance for prevention and intervention programs targeting adolescent mental health problems.

### Electronic supplementary material

Below is the link to the electronic supplementary material.

Supplementary Material 1. **Additional file 1: Table S1**. Values of EI centrality indices. **Figure S1**. Confidence intervals around edge weights for T1-network (a) and T2-network (b). **Figure S2.** Edge weight difference tests for T1-network (a) and T2-network (b). **Figure S3**. Centrality difference tests for EI for T1-network (a) and T2-network (b). **Figure S4.** Temporal network containing autoregressive paths. **Figure S5.** Confidence intervals around edge weights for temporal network. **Figure S6.** Edge weight difference tests for temporal network. **Figure S7.** Centrality difference tests for in-prediction (a for cross-lagged, c for cross-construct) and out-prediction (b for cross-lagged, d for cross-construct)

Supplementary Material 2

## Data Availability

The datasets used and/or analyzed during the current study are available from the corresponding author on reasonable request.

## Abbreviations

CLPN: Cross-lagged panel network
SD: Standard deviation
CDI: Children’s Depression Inventory
STAI: State-Trait Anxiety Inventory
YSR: Youth Self-Report Scale
GGM: Gaussian graphical model
LASSO: Least absolute shrinkage and selection operator
EI: Expected impact
CIs: Confidence intervals
CS: Correlation stability
