# Supplementary material for: Network analysis of maternal parenting practices and adolescent mental health problems: a longitudinal study
Source: Child Adolesc Psychiatry Ment Health. 2024 Mar 19;18:38. doi: 10.1186/s13034-024-00728-w (PMC10953267; doi:10.1186/s13034-024-00728-w)
Supplement: Supplementary file 1 — Supplementary Material 1. Additional file 1: Table S1. Values of EI centrality indices. Figure S1. Confidence intervals around edge weights for T1-network (a) and T2-network (b). Figure S2. Edge weight difference tests for T1-network (a) and T2-network (b). Figure S3. Centrality difference tests for EI for T1-network (a) and T2-network (b). Figure S4. Temporal network containing autoregressive paths. Figure S5. Confidence intervals around edge weights for temporal network. Figure S6. Edge weight difference tests for temporal network. Figure S7. Centrality difference tests for in-prediction (a for cross-lagged, c for cross-construct) and out-prediction (b for cross-lagged, d for cross-construct) [file 13034_2024_728_MOESM1_ESM.docx]

**Appendices**

**Table S1. Values of EI centrality indices**

| Node | T1 | T2 | Average |
| --- | --- | --- | --- |
| Aggression | 1.61 | 1.22 | 1.42 |
| Warmth | 1.20 | 1.03 | 1.12 |
| Depression | -0.17 | 0.85 | 0.34 |
| Anxiety | 0.08 | 0.40 | 0.24 |
| Inductive Reasoning | 0.33 | 0.00 | 0.16 |
| Conduct Problem | 0.05 | 0.12 | 0.08 |
| Hostility | -0.75 | -0.87 | -0.81 |
| Harshness | -0.66 | -1.31 | -0.98 |
| Monitoring | -1.68 | -1.44 | -1.56 |


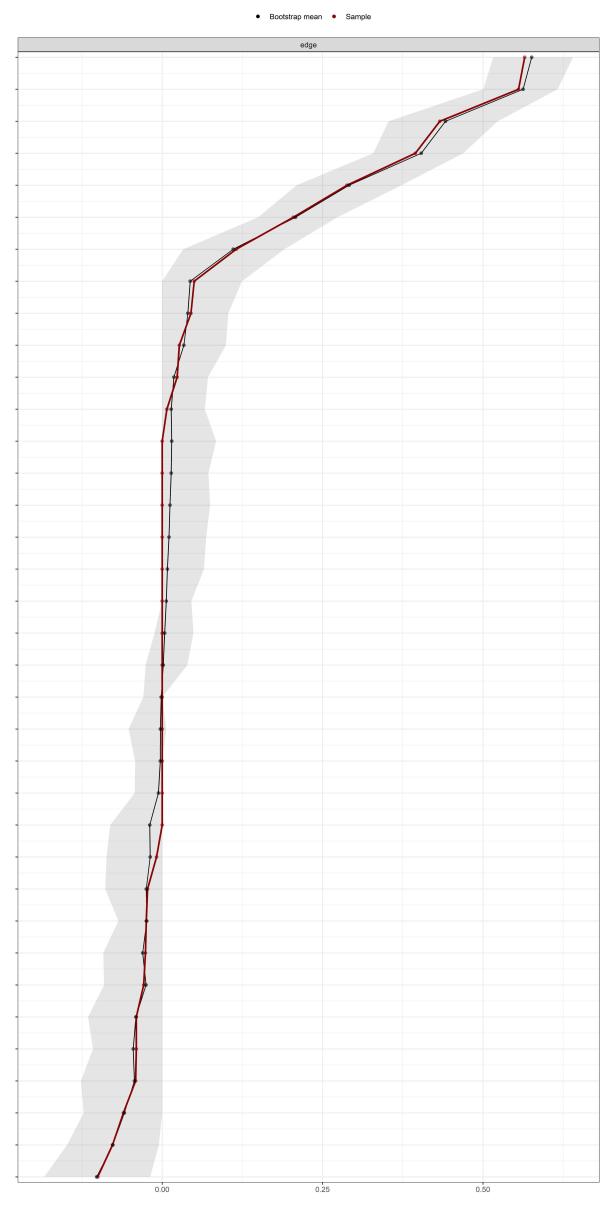

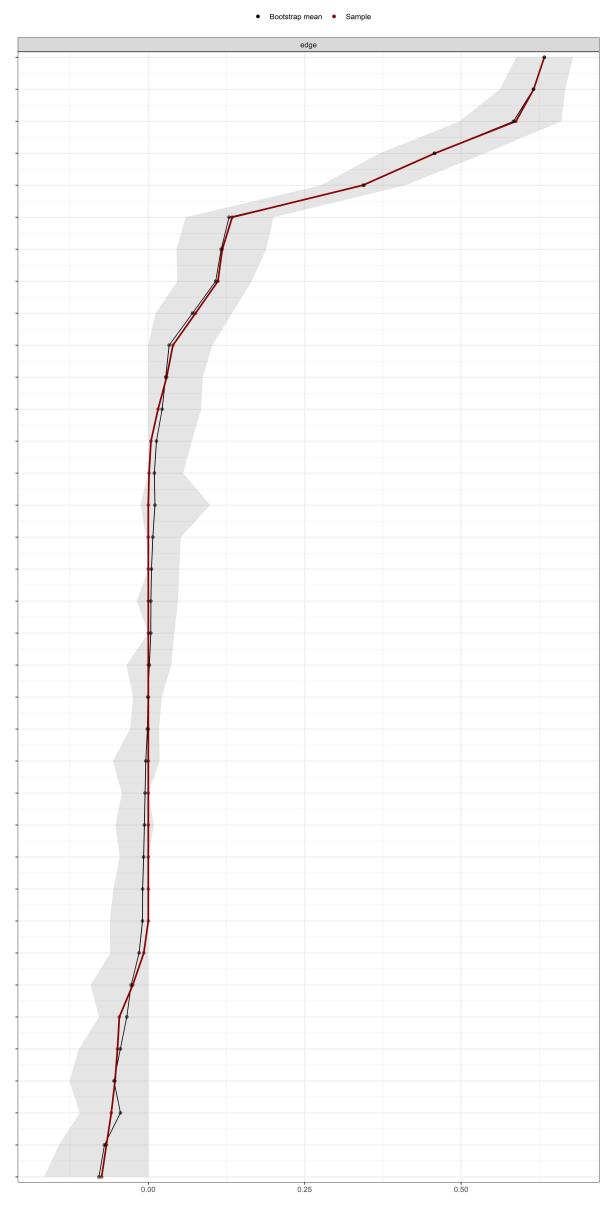


a b

**Figure S1. Confidence intervals around edge weights for T1-network (a) and T2-network (b).**


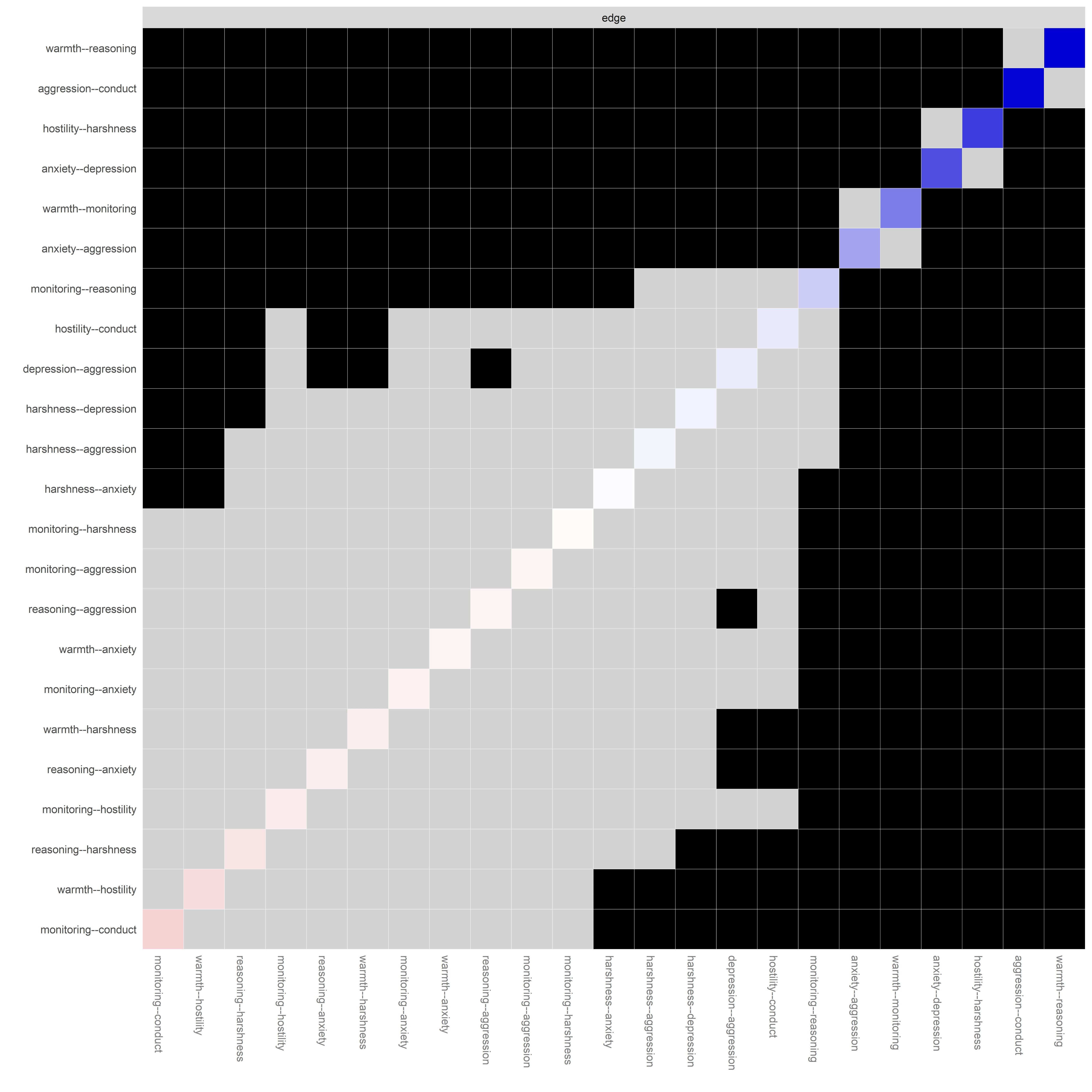


a


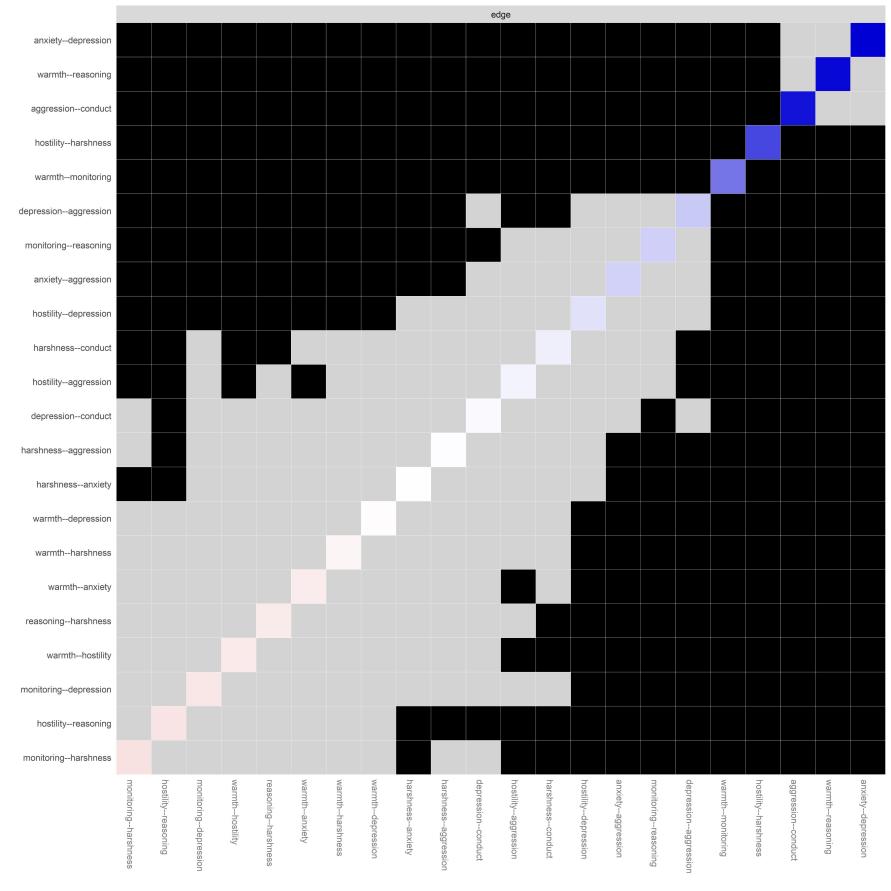


b

**Figure S2. Edge weight difference tests for T1-network (a) and T2-network (b).**

**
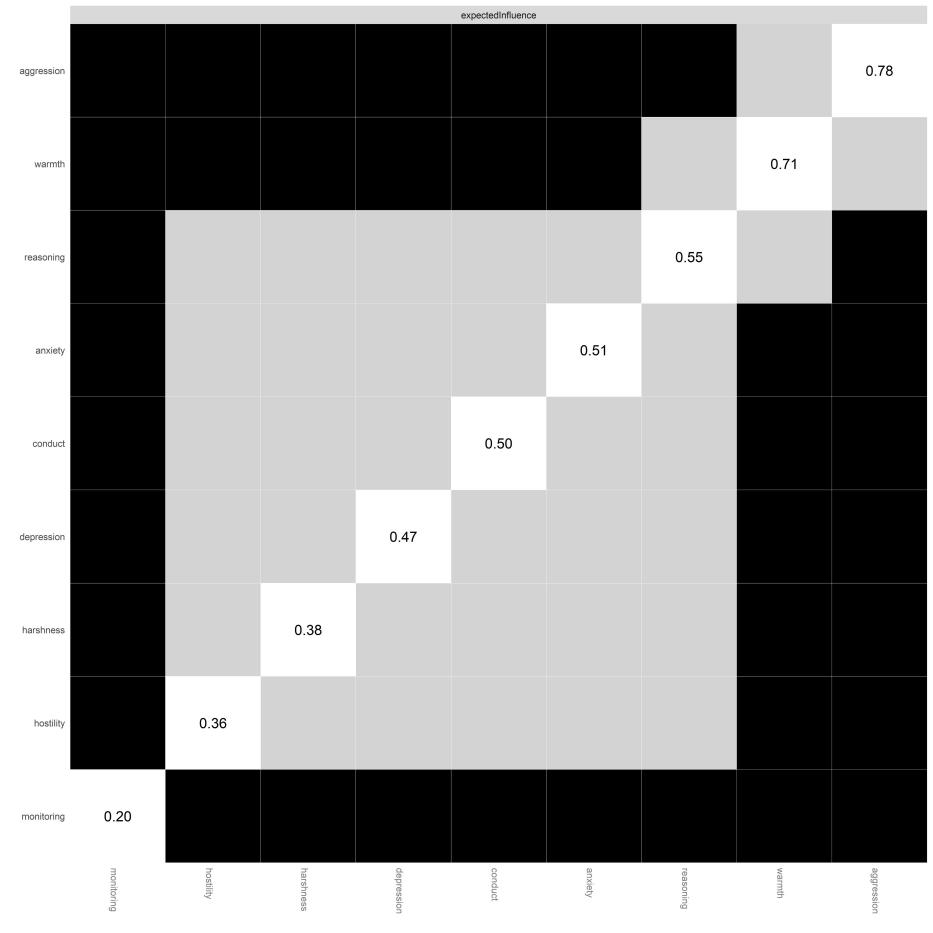
**

a

**
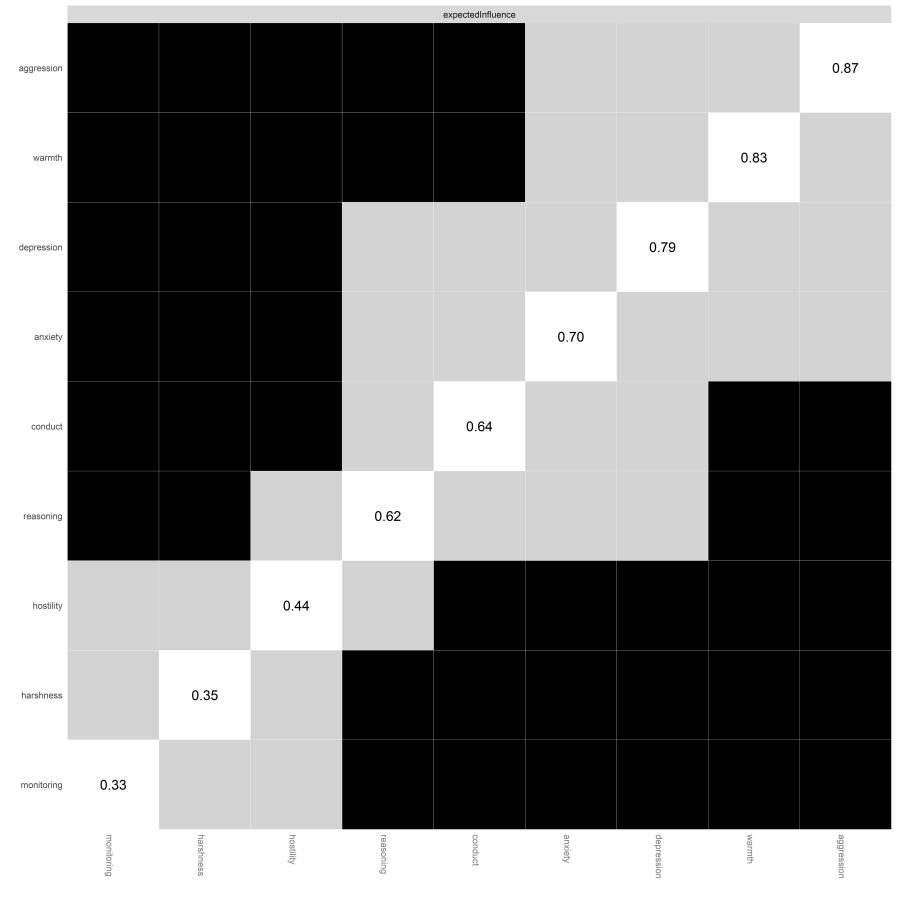
**

b

**Figure S3. Centrality difference tests for EI for T1-network (a) and T2-network (b).**

**
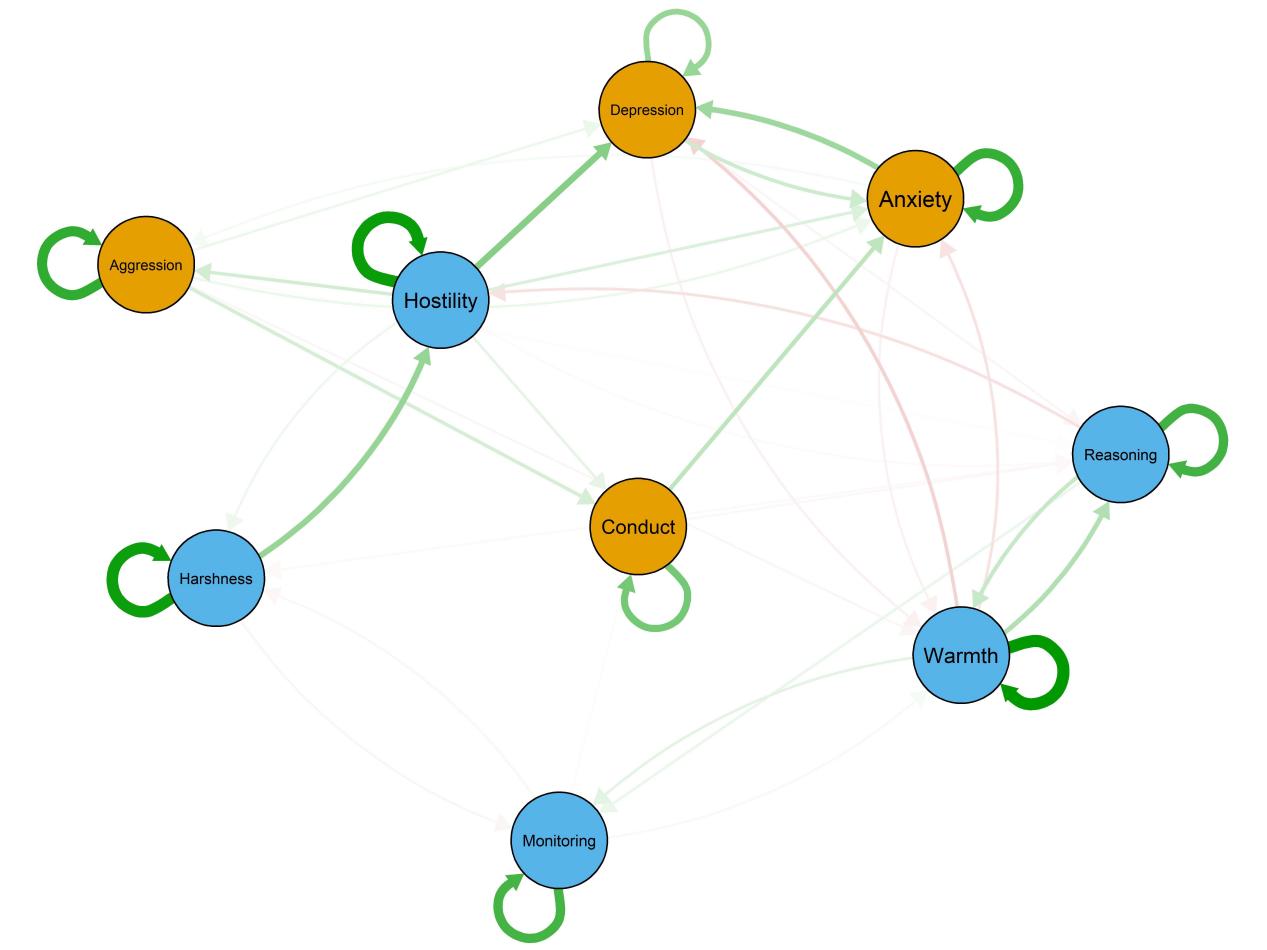
**

**Figure S4. Temporal network containing autoregressive paths.**

**
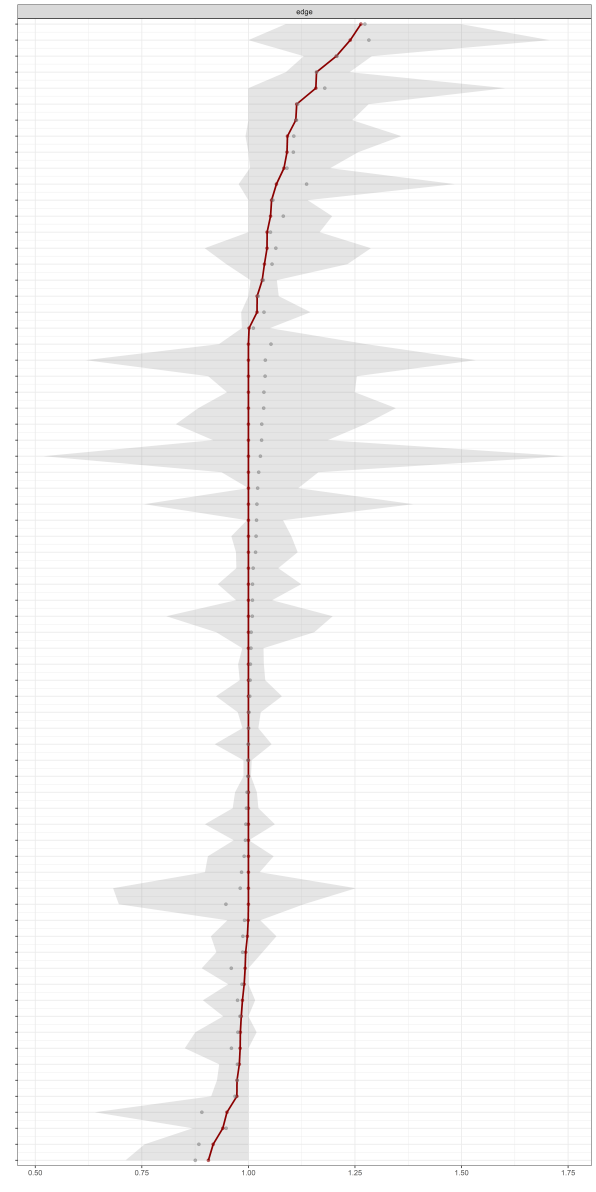
**

**Figure S5. Confidence intervals around edge weights for temporal network.**


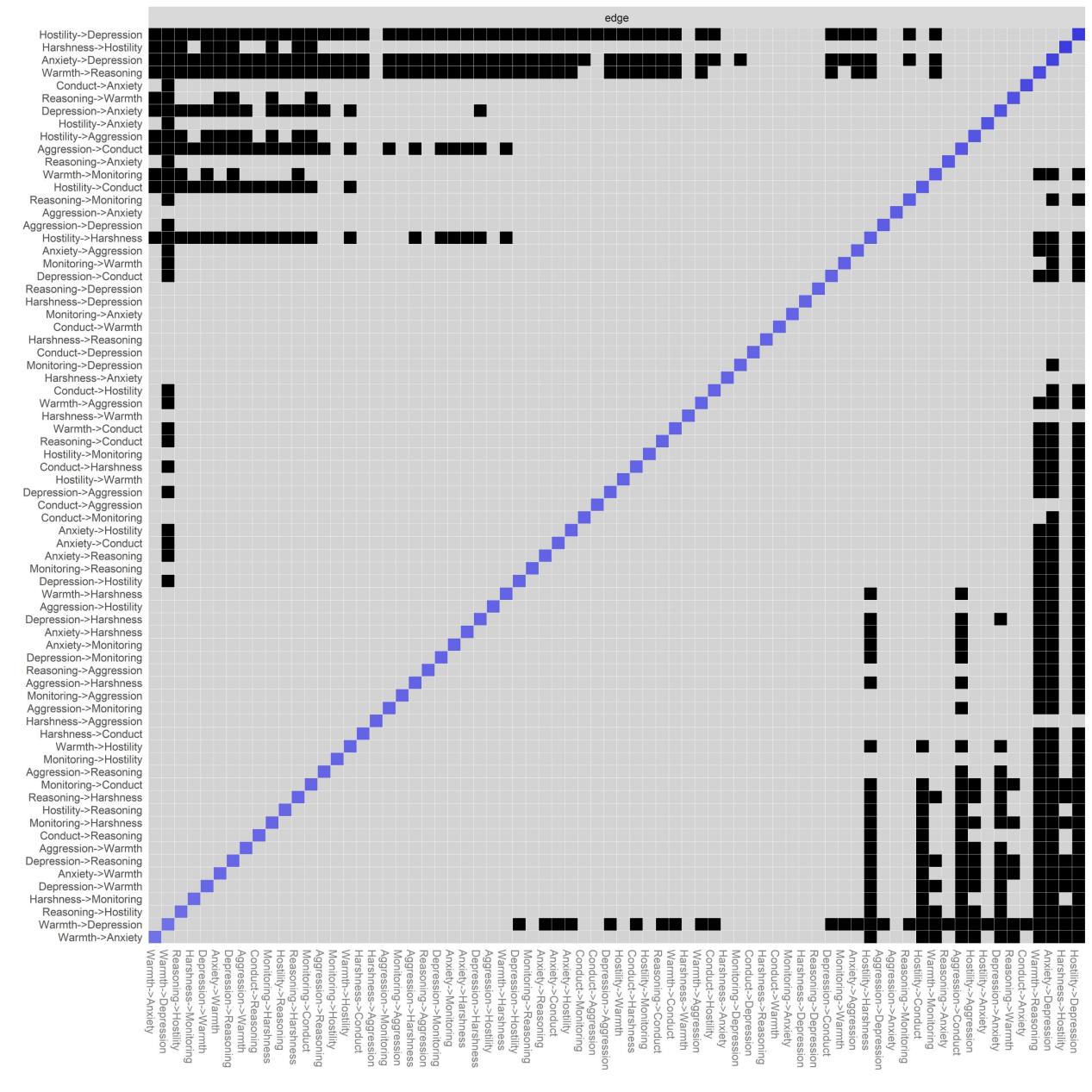


**Figure S6. Edge weight difference tests for temporal network.**


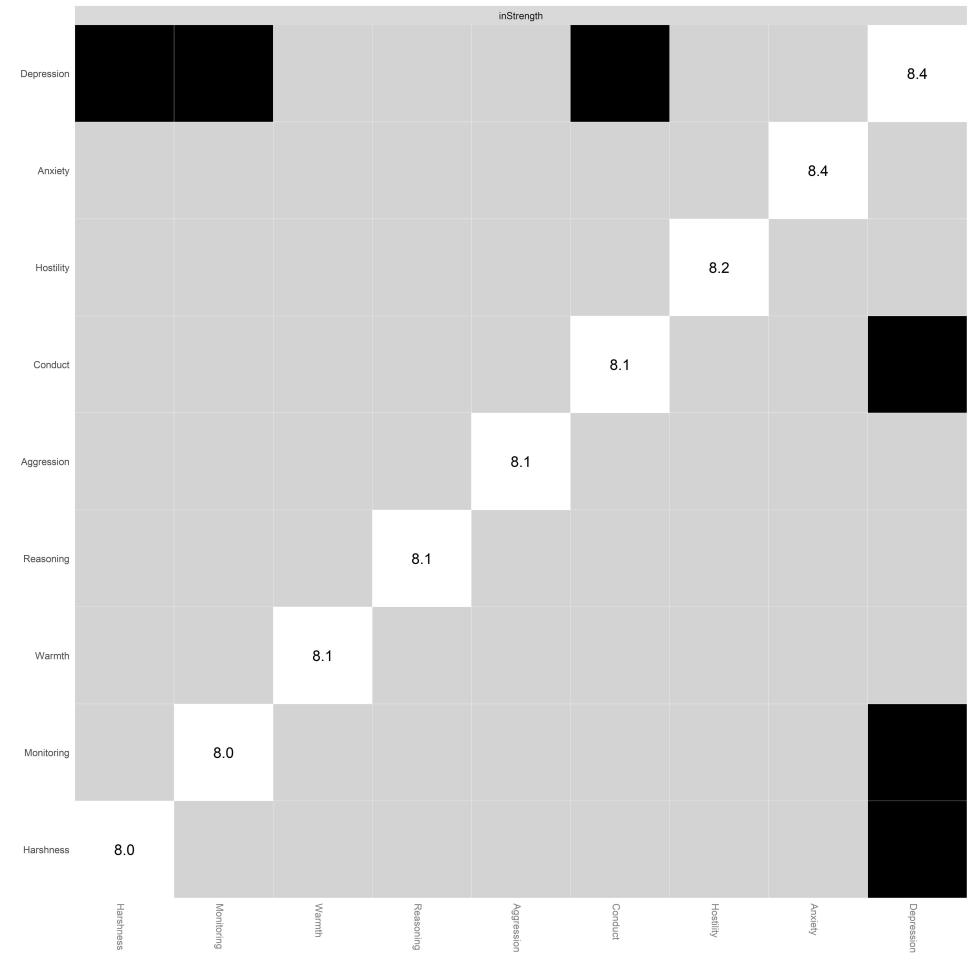


a


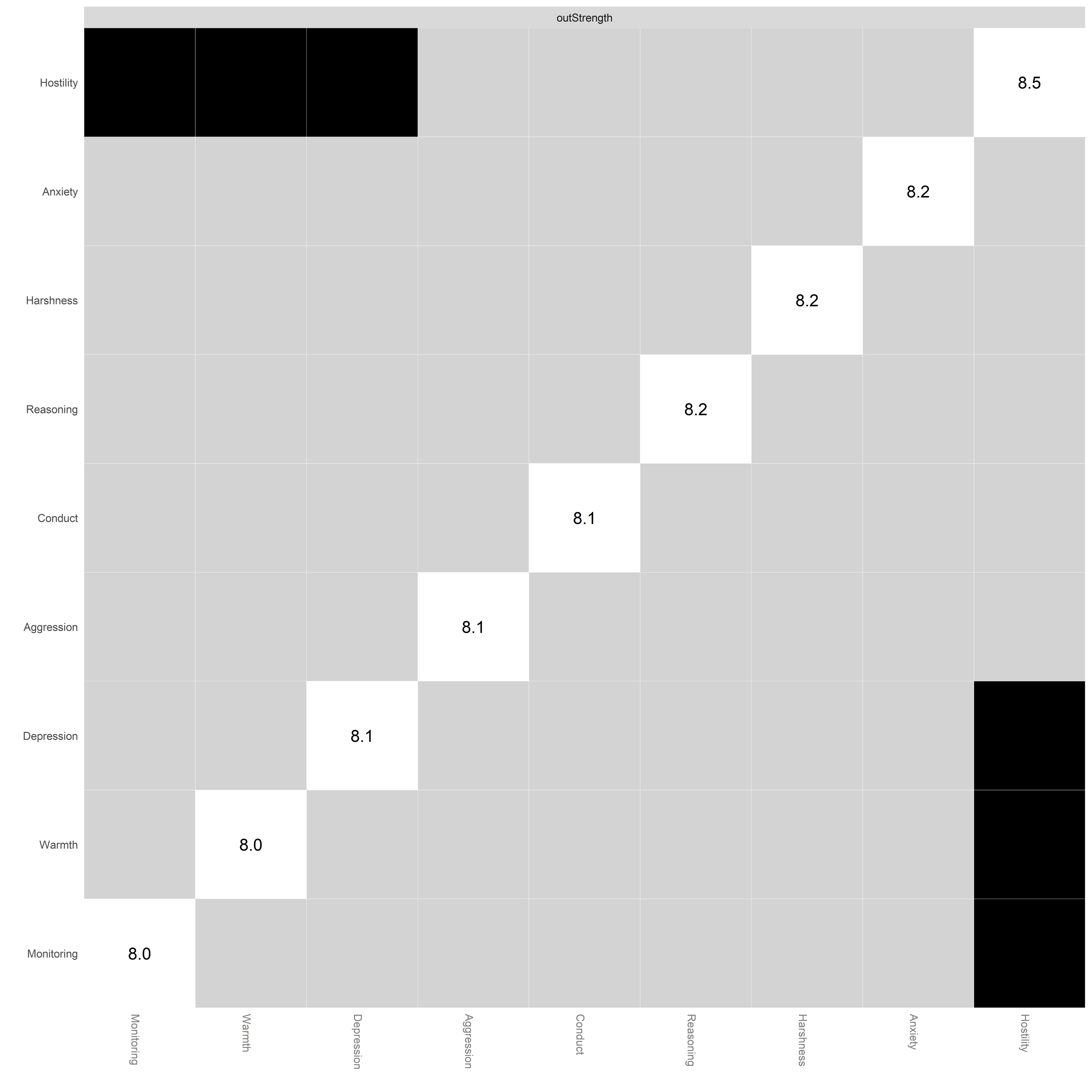


b


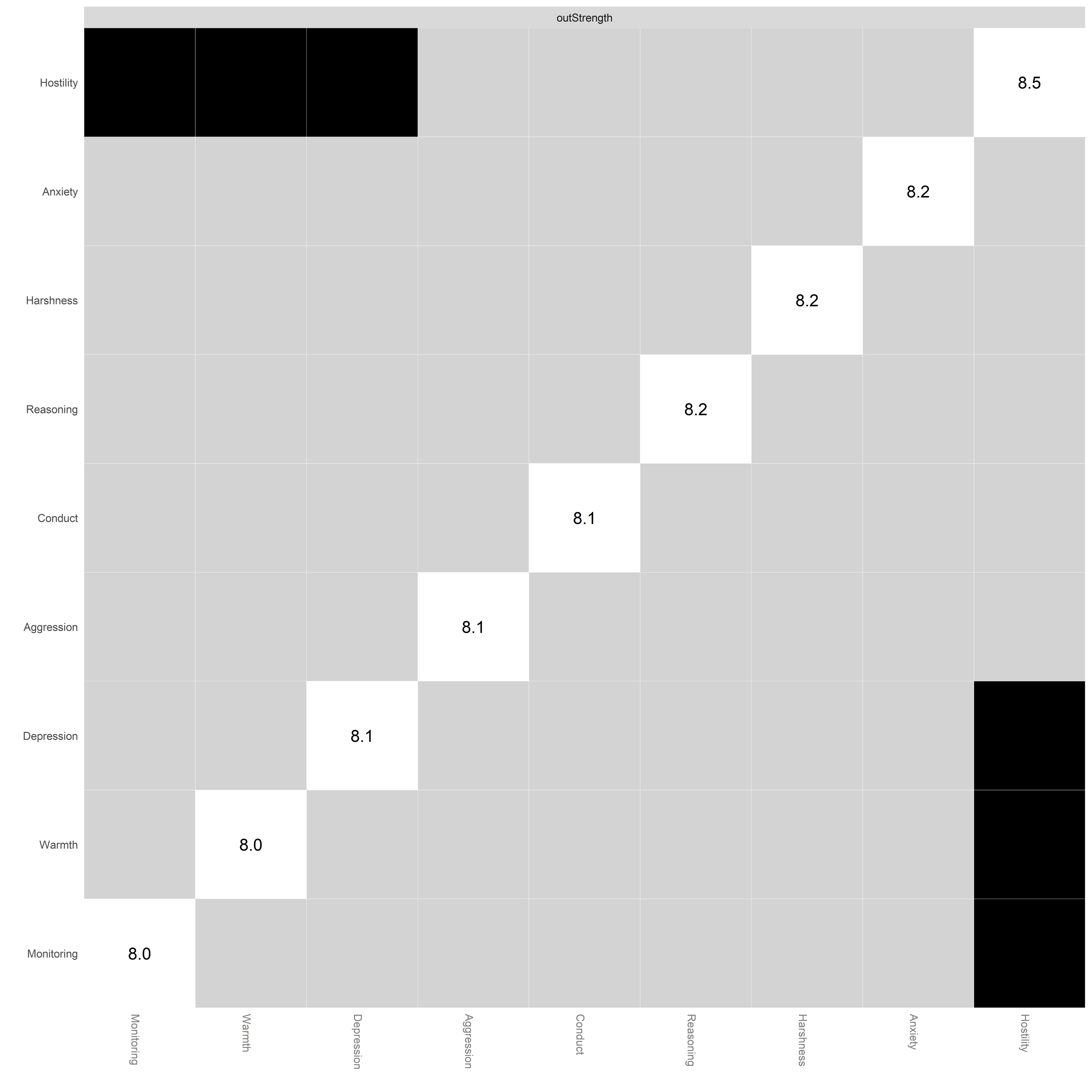


c


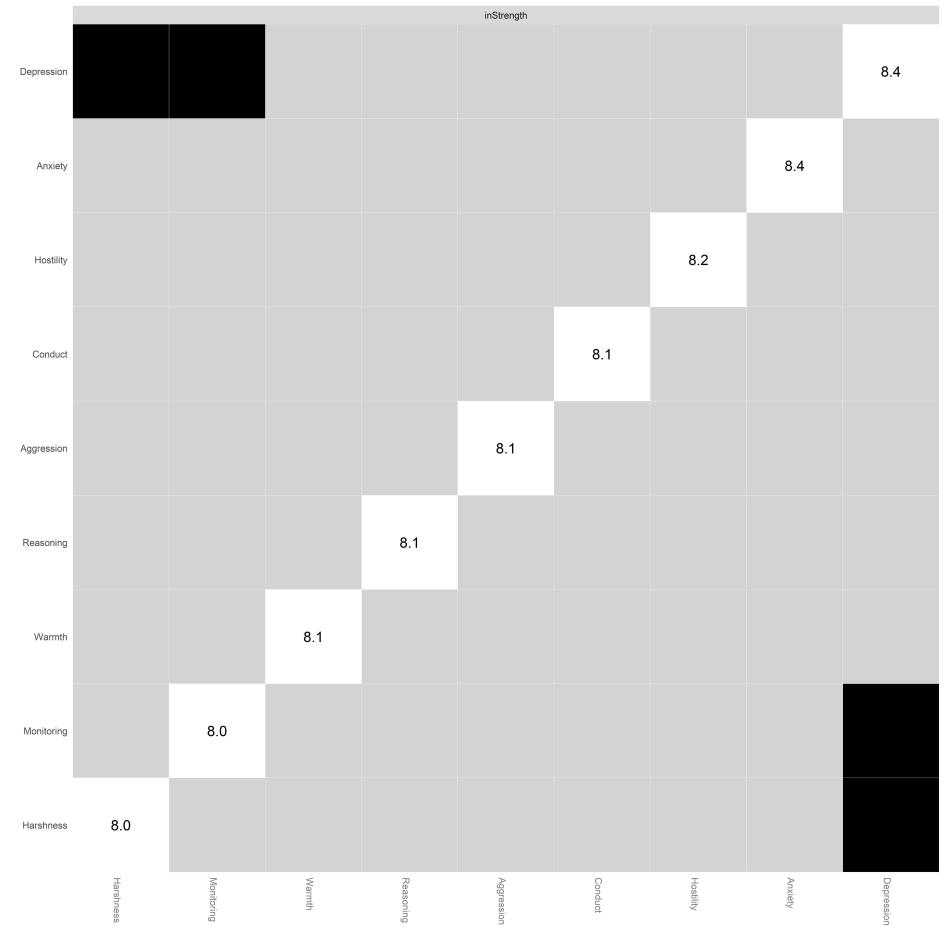


d

**Figure S7. Centrality difference tests for in-prediction (a for cross-lagged, c for cross-construct) and out-prediction (b for cross-lagged, d for cross-construct).**
