# Supplementary material for: Network analysis of maternal parenting practices and adolescent mental health problems: a longitudinal study
Source: Child Adolesc Psychiatry Ment Health. 2024 Mar 19;18:38. doi: 10.1186/s13034-024-00728-w (PMC10953267; doi:10.1186/s13034-024-00728-w)
Supplement: Supplementary file 2 — Supplementary Material 2 [file 13034_2024_728_MOESM2_ESM.doc]

**Appendices**

***Table S1 Values of EI centrality indices***

| Node | EI | | |
| --- | --- | --- | --- |
|  | T1 | T2 | Average |
| Warmth | 1.22 | 1.12 | 1.17 |
| Monitoring | -1.75 | -1.46 | -1.61 |
| Hostility | -0.65 | -0.85 | -0.75 |
| Inductive reasoning | 0.32 | -0.02 | 0.15 |
| Harshness | -0.62 | -1.22 | -0.92 |
| Anxiety | -0.08 | 0.42 | 0.17 |
| Depression | -0.12 | 0.77 | 0.33 |
| Aggression | 1.59 | 1.28 | 1.44 |
| Conduct problem | 0.08 | -0.04 | 0.02 |

EI Expected impact


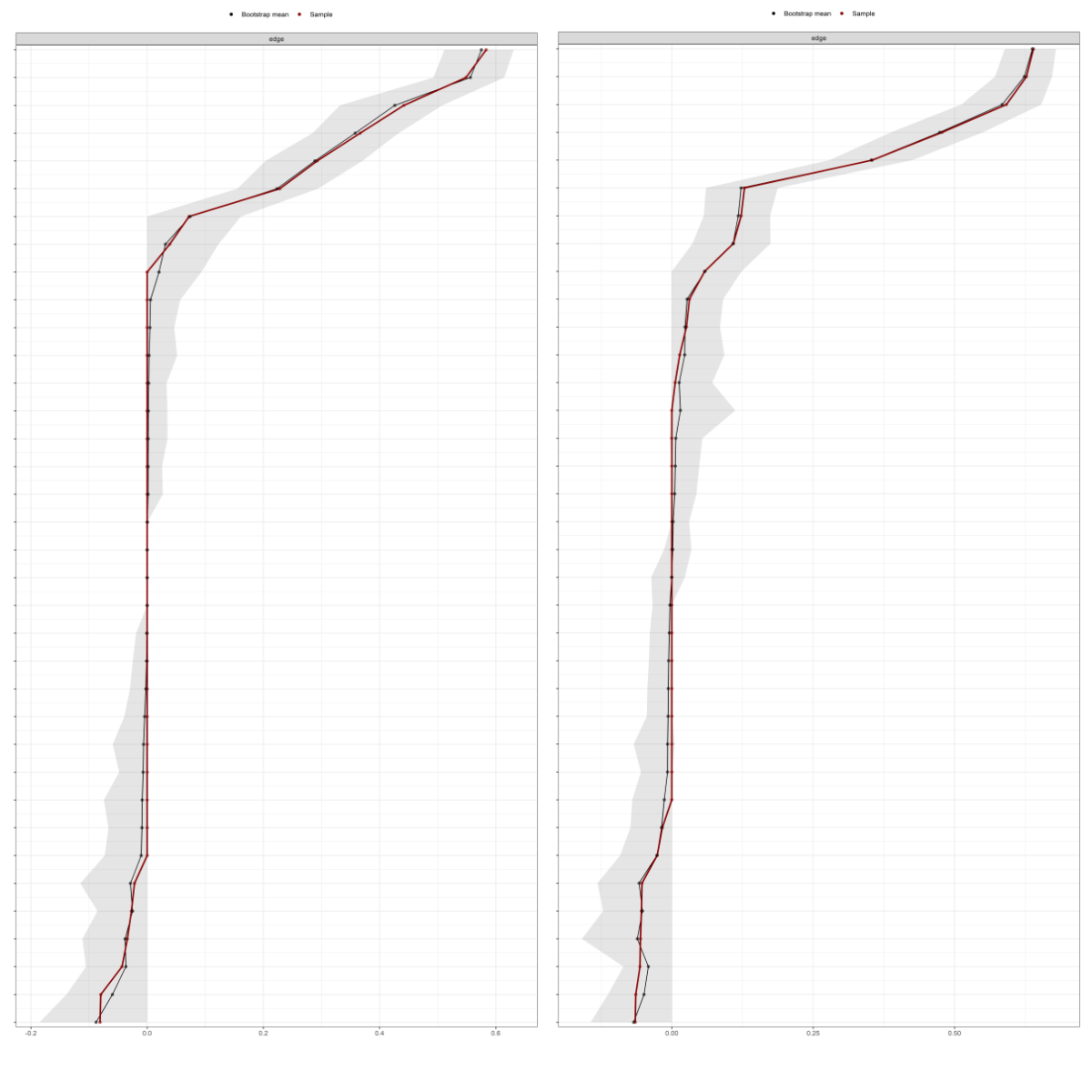


**Figure1 S1: Confidence intervals around edge weights for T1-network (left) and T2-network (right).**


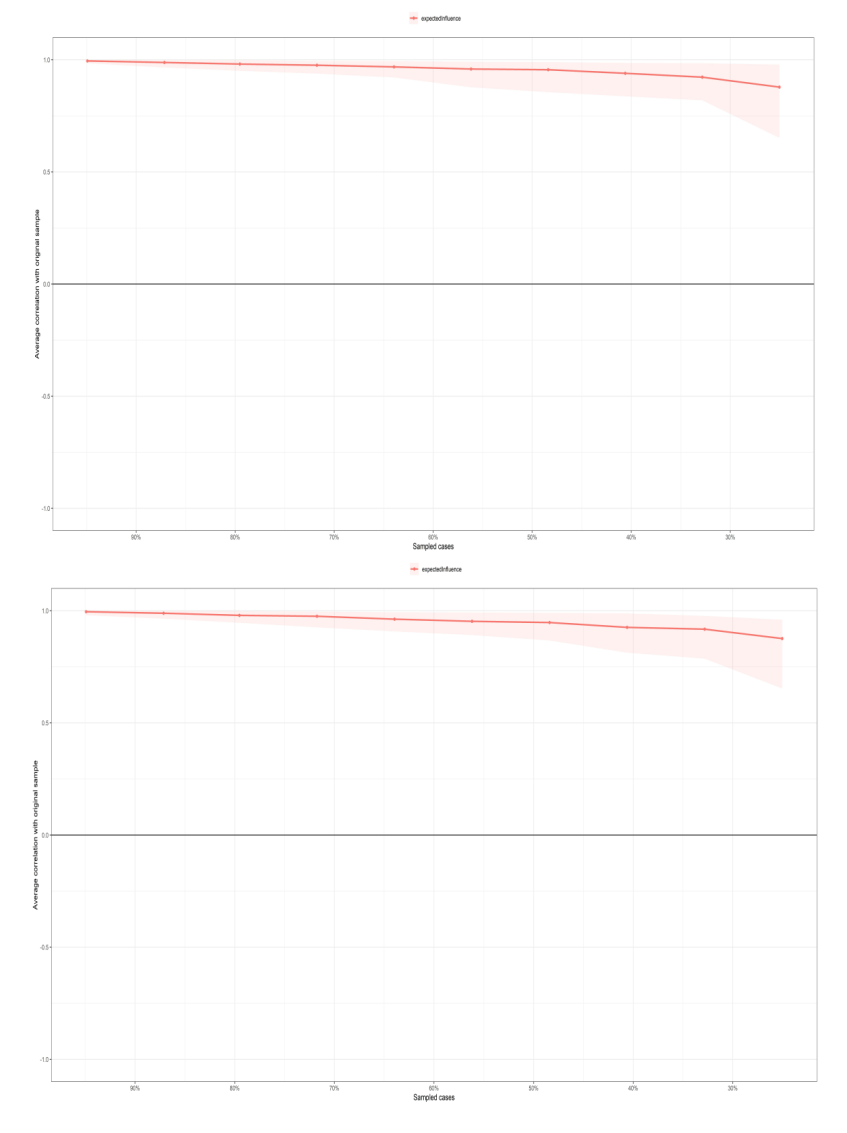


**Figure S2:** **Stability of centrality measures for T1-network (up) and T2-network (down).**


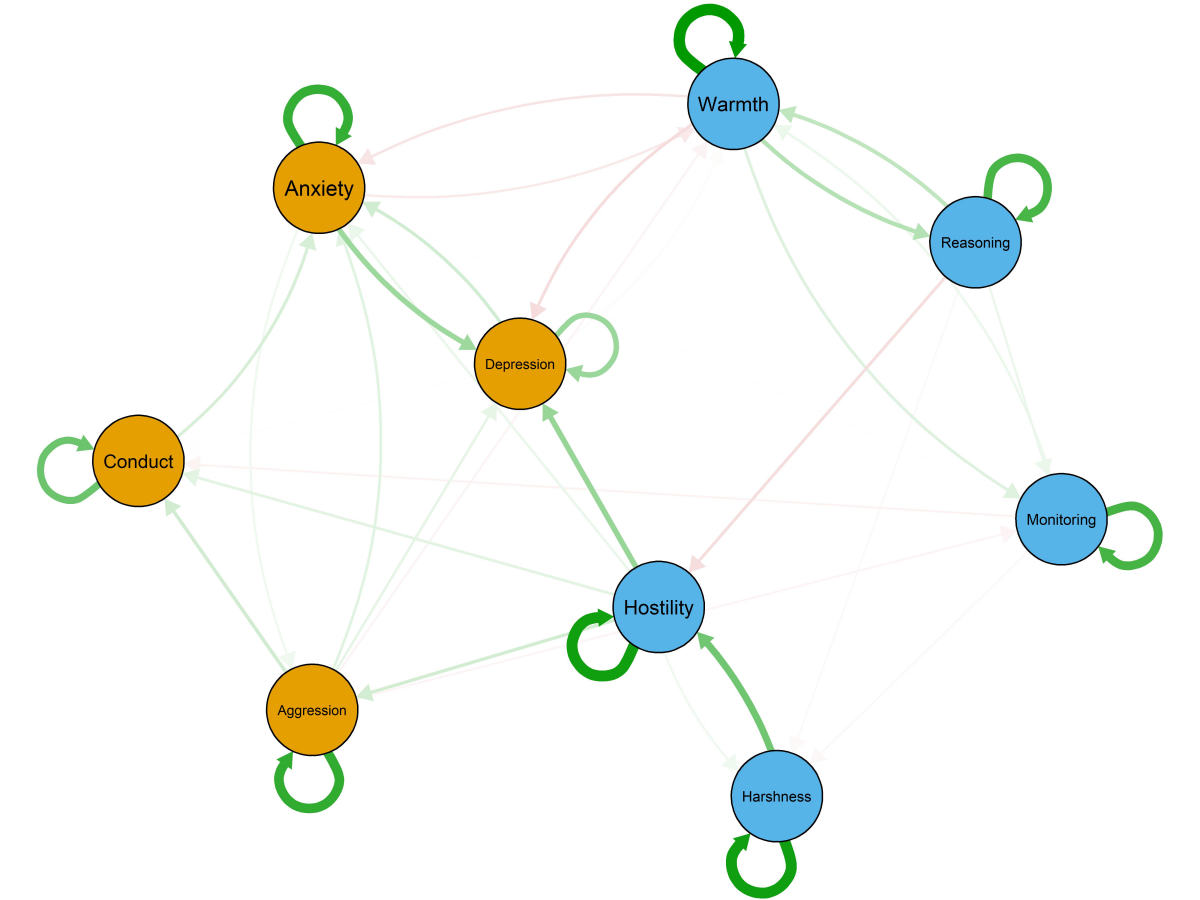


**Figure S3: Temporal network containing autoregressive paths.**


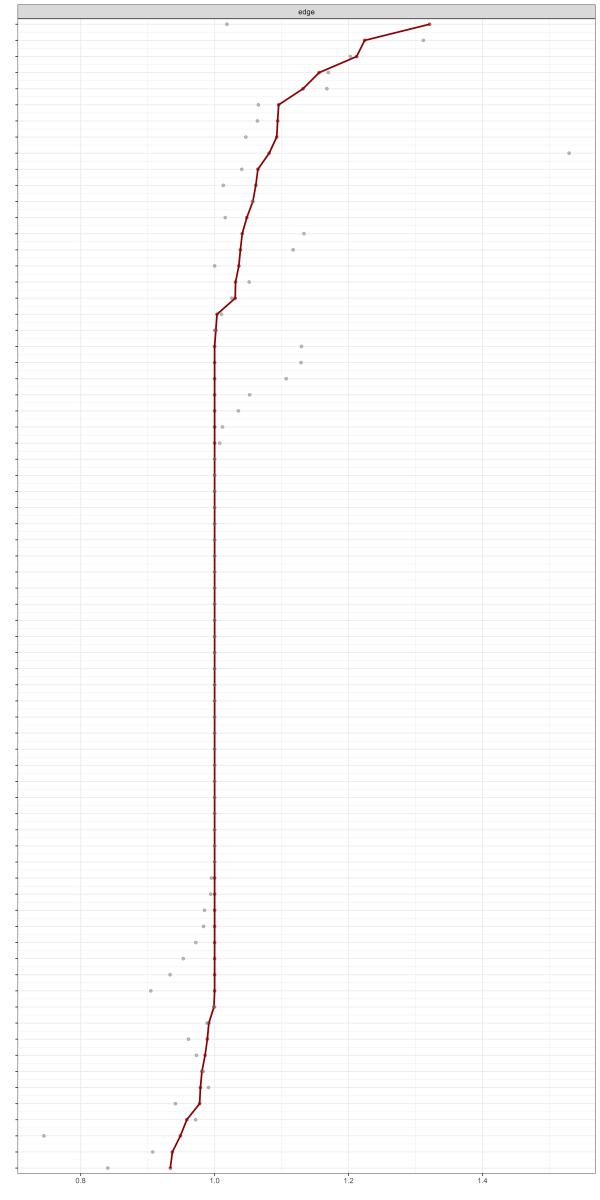


**Figure S4: Confidence intervals around edge weights for temporal network.**


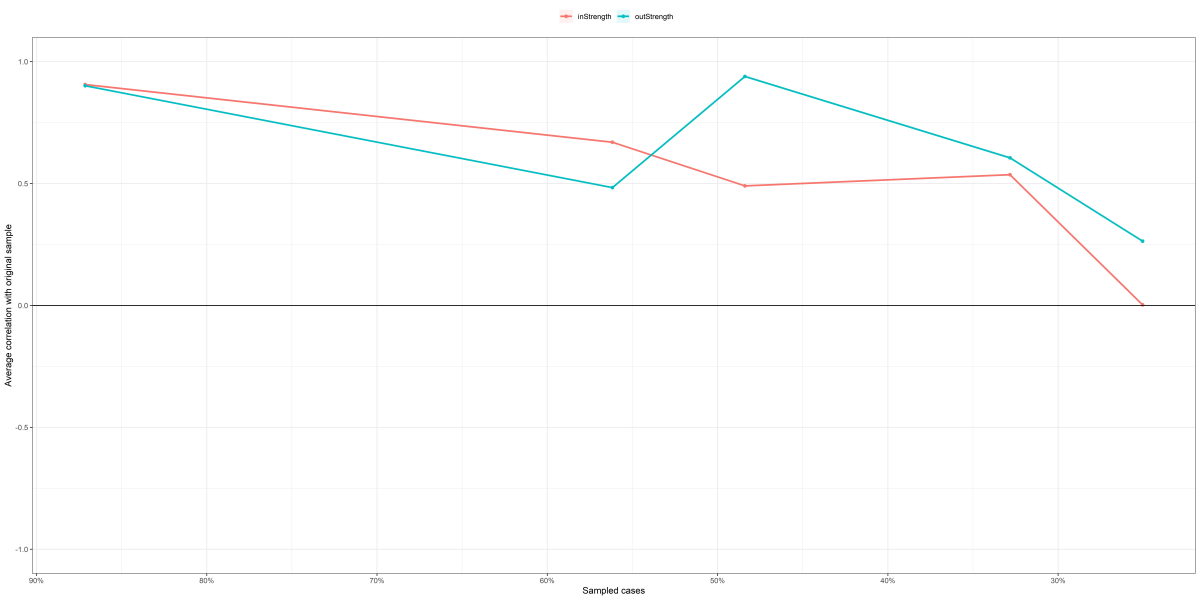


**Figure S5: Stability of centrality measures for temporal network.**
